# Patients’ perception on quality of care for prostate cancer at tertiary hospitals in Tanzania: a qualitative study

**DOI:** 10.3389/fonc.2024.1492302

**Published:** 2024-12-09

**Authors:** Obadia Nyongole, Deodatus Kakoko, Nathanael Sirili, Gasto Frumence, Daudi Simba, David Urassa, Bruno Sunguya

**Affiliations:** ^1^ Department of Surgery, Muhimbili University of Health and Allied Sciences, Dar es Salaam, Tanzania; ^2^ Department of Community Health, Muhimbili University of Health and Allied Sciences, Dar es Salaam, Tanzania; ^3^ Department of Behavioral Sciences, Muhimbili University of Health and Allied Sciences, Dar es Salaam, Tanzania; ^4^ Department of Development Studies, Muhimbili University of Health and Allied Sciences, Dar es Salaam, Tanzania

**Keywords:** prostate cancer, quality of care, treatment delays, perception, organization of services

## Abstract

**Background:**

Prostate cancer is a common cancer among men globally and its treatment affects quality of life. Poor patients’ perception of prostate cancer services may lead to their late presentation for care, often presenting with the advanced stage of the disease. This may vary from one region to another.

**Objective:**

This study, therefore, aimed to explore patients’ perceptions of the quality of care for prostate cancer in Tanzania.

**Methods:**

This qualitative case study was conducted in five tertiary hospitals in Tanzania in November 2023. In-depth interviews were conducted with prostate cancer patients on treatment for not less than 6 months. Data on patients’ perception of prostate cancer, quality of prostate cancer services, and quality of life among prostate cancer patients were collected. Thematic analysis used a hybrid inductive and deductive reasoning approach through NVivo 14 software.

**Results:**

A total of 17 interviews were conducted, resulting in four themes. These were perception of prostate cancer on the causes and diagnostic methods, delays of care leading to late presentation, perception of quality of life after treatment for prostate cancer recovery versus cancer progression, and quality of service in terms of organization of service delivery for prostate cancer at tertiary hospitals in Tanzania.

**Conclusion:**

Quality of services for prostate cancer was well-perceived, especially communication for psychosocial support. A good perception of the quality of service was found to influence the uptake of prostate cancer services. Prostate cancer patients have mixed perceptions about the quality of life after treatment, which delays health seeking, leading to late presentation. Despite efforts in awareness campaigns through different platforms, more effort is needed in determining the causes of prostate cancer, the diagnostic/screening methods necessary for prostate cancer, timely health seeking, the available treatment options for prostate cancer, and the expected quality of life after treatment.

## Introduction

Prostate cancer (PC) is a common cancer in men globally (1). Tanzania is no exception (1). The country reported a total of 40,464 new cancer cases and 26,945 deaths in 2020 (1). Poor perception of cancer; delays in seeking healthcare, reaching hospitals, and receiving care; and low healthcare utilization fuel mortality rates in men with PC (2–4). Among African men, knowledge and perception of PC services remain low and are worsened by traditional beliefs, taboos, and community stigma towards the disease and people suffering from the disease (5–8).

PC treatment affects quality of life even though it needs a holistic approach for a better prognosis (9). Improving the health system around cancer care can improve treatment outcomes (4). This can be achieved by understanding patients’ experiences to enhance linkages between inputs, outputs, and outcomes for service improvement (3, 9–11). A balance in the health system building blocks and patients’ expectations is required for sustainable quality services provision (12–15). The uptake of services also depends on what is known and, importantly, what is communicated to the patient, and poor communication for PC may influence the low uptake of cancer services as documented in Tanzania (14).

Incorporating patients’ values and preferences in PC care is important for good patient-reported outcomes (16). Knowing patients’ perceptions of the quality of services they receive is essential for increasing patients’ involvement in their care (10). Although perceptions may vary from context to context, their understanding remains crucial for tailored interventions. In Tanzania, evidence is scarce on patients’ perception of PC services based on their experience with treatment pathways (17, 18). This study aimed to explore patients’ perceptions of the quality of care for PC in Tanzania.

## Methods

### Study design

This explanatory qualitative case study (19–21) examined patients’ perceptions of PC services in Tanzania.

### Study settings

The study was conducted in five tertiary hospitals that are capable of managing and caring for PC in Tanzania. These are Ocean Road Cancer Institute (ORCI) and Muhimbili National Hospital (MNH) in the Eastern zone, Mbeya Zonal Referral Hospital (MZRH) in the Southern Highlands, Kilimanjaro Christian Medical Center (KCMC) in the Northeastern zone, and Bugando Medical Center (BMC) in the Lake zone. Three tertiary hospitals (MNH, ORCI, and MZRH) are public facilities, while KCMC and BMC are owned by faith-based organizations. They all receive patients referred from lower-level facilities and have different payment mechanisms including health insurance, out-of-pocket payment, or patients’ exemption by policy. These facilities can provide oncology and urology services (22, 23).

### Study population

Patients with PC who have been on treatment for not less than 6 months were eligible to participate in this study. A purposive sampling strategy was used to select PC patients who had consented to participate.

### Data collection tool

Semi-structured interview guides were developed in English and translated to Swahili for in-depth interviews (IDIs) regarding the patient’s perception of PC services and experience of the treatment pathway. The interview guides were reviewed and pretested through a consultative process involving experts in qualitative research. The research team consisted of five experienced health professionals including a urologist who has been attending patients with PC with little knowledge of qualitative research, a medical doctor, two nurses, and one public health specialist with experience in conducting qualitative studies. The research team was trained on the study objectives and ethics so that prior assumptions and beliefs could not influence the perception or interpretation of findings.

### Data collection

The data were collected in November 2023. IDIs lasted approximately 15–30 min each and were conducted by the first author assisted by one experienced qualitative research assistant. The interviews were conducted in Kiswahili, a common language spoken by a majority of Tanzanians.

### Recruitment of participants

Patients with PC were recruited for IDIs purposefully based on the willingness to participate in the study and also the duration of treatment for PC. Nurses and urologists/oncologists working at urology or oncology clinics of the respective hospitals were informed by hospital administration about the study. The research team explained the purpose of the study and requested for the required support from participants based on the inclusion criteria. Recruitment of study participants was interactive based on the schedule of clinics in which the clinic staff could call the research team upon receiving the patients who meet study criteria taking into consideration the participants’ values.

### Conduct of interviews

They were conducted in consultation rooms within the urological or oncology outpatient clinics. We used a digital audio recorder for the interviews after obtaining informed consent. Moderation and field notes were taken by the principal investigator and one member of the research team.

### Data management and quality control

We used verbatim transcription of the IDIs before coding. The transcripts were translated from Swahili to English. For data security, we converted recorded information into a code using a free tool for encryption to prevent unauthorized access, protected with a password, and stored in a locked cabinet before and after analysis. Only the principal investigator and one research assistant had access to the data. Participants and the five tertiary hospitals were assigned numbers for anonymity. Participants from each hospital were given a range of numbers.

### Data analysis

We adopted the six stages of thematic analysis employing both deductive and inductive approaches (24–26). A line-by-line coding of the interviews was done to assist the ownership of the data including making sense of patterns, connections, and the bigger picture from the data by the principal investigator. Quality check was done by one research assistant who is a nurse specialist experienced in qualitative research (27). Further analysis involved collapsing multiple codes that share a similar underlying concept or feature of the data into one single code (25). A code that turned out to be representative of an overarching narrative within the data was promoted to a sub-theme or even a theme. We used NVivo 14 software in the analysis to generate codes, sub-themes, and themes.

## Results

A total of 17 interviews were conducted with participants aged 50 years and above with a median age of 64 years. Ten participants had primary school education. Eleven participants were retired civil servants and 13 were married. Some participants were on treatment for PC for more than 4 years as summarized in **Table 1**.

**Table 1:** Demographic characteristics of participants.

Characteristics	Frequency
Age (in years)	
50–60	4 (23.5%)
61–70	9 (52.9%)
71–80	3 (17.6%)
>80	1 (5.9%)
Education	
Never went to school	1 (5.9%)
Primary education	10 (58.8%)
Secondary education	3 (17.6%)
University education	3 (17.6%)
Marital status	
Married	13 (76.5%)
Not married	1 (5.9%)
Widowed	3 (17.6%)
Employment status	
In service	3 (17.6%)
Retired	11 (64.7%)
Peasants	3 (17.6%)

### Summary of findings

Four themes emerged as a result of responding to questions regarding the understanding, perception, and real experience of PC patients. They were linked to causes of late presentation for PC care, namely, perception of PC, delays of care, perception of quality of life after treatment, and quality of service for PC at tertiary hospitals in Tanzania (**Table 2**).

**Table 2:** Codes, sub-themes, and themes.

Codes	Sub-themes	Themes
Prostate cancer affects elderly people	Perception regarding people at risk of prostate cancer	Patients’ perceptions of prostate cancer
Prostate cancer is a terminal disease	Perception of prostate cancer fatality	
Prostate cancer is due to witchcraft	Perception on causes of prostate cancer Perceptions and attitudes on prostate cancer diagnosis methods	
Prostate cancer is due to sexual promiscuity Blood test Finger test Fear of positive results		
Traditional care	Sources of healthcare	Delays of care for prostate cancer
Hospital care		
Delayed care seeking Delayed results	Timing of seeking care Timeliness of results	
Reduction/loss of sexual function	Negative perceptions of prostate cancer treatment outcomes	Perception of treatment and quality of life after treatment for prostate cancer
Hormonal and radiation side effects of treatment		
Improvements in health condition	Positive perceptions of prostate cancer treatment outcomes	
Relieve symptoms after treatment		
Reduced stigma		
Good communication	Positive experiences of care for prostate cancer patients	Perceptions of quality of healthcare to patients with prostate cancer
Provider–patient interaction for psychosocial support		
Provision of patient education		
Long waiting time	Negative experiences of care to prostate cancer patients	
Delays in referral		
Misdiagnosis		
Fragmented services Inability to afford the cost Access to providers and services		

### Patients’ perceptions of prostate cancer

Participants were living with PC as part of aging because they perceived it as a disease of elderly people, and they were waiting to die because they knew cancer had no cure. Expectedly, the perception that PC is caused by witchcraft was reported. As a result, patients were treated by traditional healers before seeking hospital care, causing a further delay in receiving effective treatment. Some patients thought that PC is caused by indulging in promiscuity. These perceptions favored self-stigma from the community and late presentation to hospitals. The quotes below reflect the patients’ perception of possible causes of PC.

Prostate cancer is a disease of elderly men.

*“I have been hearing that elderly people get cancer and die”* (Participant 1-01).

There is no cure for prostate cancer.

*“… I know that once you get cancer you die because there is no cure”* (Participant 5-14).

Traditional healers can treat prostate cancer.

*“If you are bewitched only traditional healers can treat”* (Participant 3-11).

Prostate cancer is a sexually transmitted disease.

*“I think prostate cancer is caused by having multiple sexual partners*” (Participant 4-08).

When asked about what they perceive as the best method for the diagnosis of PC, most of the participants were afraid of going to the hospital because they knew the finger would be inserted into their rectum for diagnosis of PC **[**digital rectal examination (DRE)]. After probing into the reasons, it was revealed that the digital rectal test is perceived to devalue the sense of manhood. Moreover, patients did not accept the DRE mostly due to self- or community stigma. Participants had a perception that once the community knew they went to the hospital for PC treatment, they would be labeled as hopeless men. Most participants reported preferring prostate-specific antigen (blood test) for the diagnosis of PC. Participants did not understand the concept of complementarity of the two tests to increase sensitivity and specificity. Participants are also worried about getting the results of cancer:

DRE is not accepted.

*“I was afraid of going to the hospital- because my friend told me they would use a digital rectal test for prostate cancer, I requested my doctor to use only blood test, not digital rectal test and to be honest I don’t know why doctors use digital test”* (Participant 1-02).

### Perception of treatment and quality of life after treatment for prostate cancer

We received mixed opinions related to quality of life after treatment for PC as supported by different quotes from participants. Some participants reported improvement after treatment. Participants who accepted the diagnosis and treatment sounded to report better improvement regardless of the stage of disease or the type of treatment. Participants were relieved from symptoms after treatment, and this made them have a positive perception of the treatment outcome:

Quality of life improves after treatment for prostate cancer.

*“I was bedridden but now I can walk to my neighbor, I have no pain and I am eating well, life goes on until when God decides to take me like others”* (Participant 5-14).

Participants reported fear of treatment for PC because they were worried about the side effects. Some participants thought they could die immediately after surgery because cancer is not supposed to be operated on. The type of treatment was reported to devalue the participant’s quality of life especially loss of libido after treatment. Divorce was reported by participants to be linked to treatment for PC, which is accompanied by reduced sexual function. This reflects a lack of sexual partner support for good treatment outcomes. Couple counseling though was not part of the finding, but here, the need cannot be underestimated.

Treatment for prostate cancer can propagate divorce.

*“I had two wives, the younger wife left me and got married to another man because she said I am functionless*” (Participant 3-09).

Participants had negative perceptions of radiotherapy and surgery for PC. This explains the loss of follow-up after prostate diagnosis due to an unwillingness to start treatment. Participant’s reticence about what is perceived by the community made them very reluctant to radiotherapy and/or surgery for PC. Participants had a certain reticence about radiotherapy and/or surgery because they were afraid that they would die early.

Negative perception on the treatment options for prostate cancer.

*“Doctor, my community knows that radiation is not specific, it burns everything in the abdomen even health organs, but surgery is not safe for cancer because once you touch cancer you die because through operation you spread it faster*” (Participant 3-11).

The occupation of participants was reported to positively influence the acceptance of treatment; one patient reported that it was easy for him to accept treatment because he is a medical doctor by profession. Couple counseling for the treatment of PC helped the acceptance of treatment, especially in those patients who are below 65 years of age.

Proper counseling is important.

*“At first, we were worried as any other human being, but as days go by, you accept the situation after counseling although it is was not easy to accept the removal of my testicles even if I was told that it will stop cancer progression”* (Participant 1-05).

### Delays of care for prostate cancer

The three causes of delays for PC were reported at different dimensions. Participants reported difficulties in healthcare seeking because hospitals with specialists are far from them, implying that there is a lack of physical access to healthcare for PC. Some participants as mentioned earlier were reluctant to seek hospital care due to negative perceptions of methods of diagnosis and treatment for PC. They further insisted that, in acute conditions, they have difficulty reaching the hospitals and they use traditional healers because they are nearby.

Use of traditional medicines causes delays of seeking hospital care for prostate cancer.

*“In my case, I was using our traditional medicine until when urine stopped coming out I had to visit the hospital and after investigations doctor told me that I had advanced prostate cancer*” (Participant 1-03).

Some patients were reported to have died in villages before they sought hospital care specifically those who were not insured because of financial difficulties (lack of access due to poor affordability). PC is a disease of men, and participants are too shy to tell their children especially their daughters that urine is not coming out. Insurance was reported to cover the cost of PC. Participants suggested insurance be given to all people including those in villages. This was perceived to increase the utilization of hospital services at the early stage of PC for better treatment outcomes.

Treatment for prostate cancer is expensive.

*“Some of my village mates are not coming to the hospital because it is expensive, and they don’t have insurance they die without treatment in the village. I wish the government could give free treatment especially for cancer and to old people*” (Participant 5-13).

Participants reported delays in receiving services due to staff shortage. Staff are working for long hours, which might reduce their efficiency. Biopsy results were delayed, which increased the anxiety of patients while waiting for their results. Patients explained that the delay in receiving services was sometimes due to a shortage of staff and limited machines. Some patients experienced delay in receiving care and died before treatment due to a long waiting list

*“You can see even here we are many in the waiting line and doctors cannot even go for lunch. I have a friend who was from Iringa, and we were waiting together to start the radiotherapy here at ORCI, but he died one month ago before his schedule of radiotherapy as you know we are many”* (Participant 1-04).

### Perceptions of quality of healthcare to patients with prostate cancer

Study participants expressed their mixed opinions on the quality of services for PC. Healthcare providers (HCPs) were mentioned as key players in providing quality service through interaction. Some participants reported receiving good support from HCPs that made them feel loved. Good communication and health education were reported by participants to be important for psychosocial support even if there are no formal counseling sessions for PC.

Good interaction.

*“Doctors and nurses treat us with love, compassion, and encouragement, in general providers have good communication although I did not receive formal counseling about prostate cancer and I think they don’t have formal session may be because they are overwhelmed*” (Participant 1-05).

The study participants did not appreciate the organization of the health system for the provision of PC. PC services were fragmented because tertiary hospitals did not provide comprehensive care for PC. This was reported to cause delays in care and increased cost of treatment. Participants reported a delay in getting services due to an uncoordinated referral system. The health information management system (HIMS) was not integrated. PC services were reported to be centralized at zonal hospitals, but still, they are not patient-centered in service provision.

Lack of patient centered services.

*“The health information system of Mbeya is not connected to that of Ocean Road Cancer Institute so I had to start new process of treatment”* (Participant 5-14).

## Discussion

The analysis of qualitative data generated four themes regarding the perception of patients on the quality of service for PC, which might reflect the observed low rate of utilization of screening/early detection of PC and late presentation in Tanzania. The themes were the perception of PC, delays of care, perception of quality of life after treatment, and quality of service for PC at tertiary hospitals in Tanzania.

A mixed perception of PC was based on an understanding of the causes of PC and the diagnostic methods that would impair health-seeking practice. Participants perceived PC as part of aging in men and having no cure. This perception contributes to poor health-seeking behavior, resulting in patients presenting with advanced PC in Tanzania as seen in this study. This perception is similar to the findings from other quantitative studies in Africa in which most patients present at late stages of cancer due to many factors related to the health system and social–cultural attributes (2). The use of traditional healers for the treatment of PC is common practice in Africa, which is sometimes due to the perception of uninformed positivists that PC is the result of being bewitched, which contributes to late presentation (4, 5, 28). Some participants felt embarrassed telling their children about their prostate status because of the notion that PC is caused by promiscuous behavior; therefore, it was difficult for them to utilize hospital facilities due to self-stigma. This is similar to the findings of other studies in the African context where it is difficult for parents to reveal their sexually related disease to their children until it is in the advanced stage and there is no alternative treatment (3, 7, 29). DRE as a screening and diagnostic method for PC is not perceived positively by participants mainly due to cultural and community beliefs that inserting a finger in the anus of men may promote homosexuality. The perception of diagnostic methods can be improved by proper community health education, which will ultimately increase the utilization of cancer for early detection (30, 31). Moreover, negative perception of the screening/diagnostic method for PC was reported to cause delays in the decision to seek hospital services as men were ashamed and scared of DRE, and some of them did not know other diagnostic methods for PC; therefore, they would visit the hospital when the situation has worsened with a feeling of uneasiness (6, 32). This is similar to some studies suggesting that one of the barriers to PC screening is the notion that the DRE is embarrassing, painful, and uncomfortable, which has been documented in African countries and ultimately causes delays in decision-making for hospital-seeking (5, 7, 32, 33).

Delays of care for PC in this study were influenced by the decision to seek care, reaching hospitals, and delays in receiving care for PC. Some participants experience delays in receiving services, ending up with having long waiting times because providers and machines are few, and hospitals are overwhelmed, leading to some patients dying before starting treatment. Misdiagnosis was reported to cause delays in the treatment for PC; patients become dependent on qualified doctors who could properly diagnose PC. Fragmented services for PC at tertiary hospitals were reported to cause delays in providing appropriate care; this is contrary to what is recommended for a patient-centered approach in the service provision for good PROs (34). In this study, participants reported delays in receiving care due to long referral systems that were not well coordinated; this is similar to findings from other studies in SSA and globally (4, 35). Participants reported difficulties accessing services for PC even if they are available because of their inability to pay for the services due to the high cost. These findings are common in LMICs in which affordability of cancer services is a challenge since cancer treatment is expensive, and this is likely to be a barrier towards universal health coverage (4, 17, 36–38).

Quality of life after treatment for PC was mainly linked to sexual function, relief in urination, and back pain in this study. Sexual function after treatment for PC was found to be a main concern to some of our participants, with most of them being worried about erectile dysfunction after treatment. Erectile dysfunction after treatment for PC has been one of the complications and its severity varies depending on the type of treatment used (39). The fear of worsening quality of life after treatment for PC was reported in other studies, discouraging health seeking at an early stage of the disease (16). Participants who got relief from their urinary problems and back pain had a positive perception of the quality of life after treatment for PC. Furthermore, the provider–patient relationship during care was reported to improve the quality of life of patients; this is similar to the findings from other studies in which good patient–provider interaction was recommended for good patient-reported outcomes (34). A good relationship between patients and HCPs plays an important role in improving the quality of life of patients with chronic illnesses like PC (18).

Perceptions of quality of healthcare to patients with PC in this study were reported in communication for psychosocial support/patient involvement and referral system not limited to the organization of PC services. Participants reported a lack of formal counseling sessions for PC and they thought counseling would help in the acceptance of the disease and treatment. Furthermore, participants declared informal counseling during interaction with HCPs, which gave them helpful information. The provider–patient interactions reflect the quality of service, especially in the aspect of psychosocial support. The relationship can encourage them to accept treatment and adhere to the follow-up clinic schedule. Patient–provider interaction is important for psychosocial support regardless of how long it takes as recommended by standards of quality of healthcare (14, 15, 30, 40, 41). In this study, similar to most LMICs, shortage of staff and infrastructure was mentioned as one of the main limitations to the provision of quality service; however, providers were interacting with patients despite their shortage, and this could mirror the quality of the services given (10, 42). Participants felt that a fragmented HIMS impaired the referral system, which made them feel abandoned especially if they were not informed. Integration of HIMS would improve the referral system as the receiving facility could get prior information about the patients, and this would improve quality of care, and the decision to refer the patient should be participatory to create ownership of the entire process.

## Strengths and limitations of the study

This study provides information on the real experience of patients with PC, which can inform policymakers on the approach towards the provision of patient-centered services. Most of our study participants (58.8%) had primary education and 5.9% never went to school; all these might serve as limitations to the details of findings (5, 28, 29) even though trustworthiness is maintained due to the pragmatic approach and multiple study sites (43). This study used an inductive and deductive hybrid approach to accommodate both positivist and constructivist perceptions for a better understanding of the dynamics of quality of service like other studies (21, 44, 45).

## Conclusions

The provision of quality services for PC needs a holistic approach supported by effective communication for psychosocial support and good organization. The uptake of PC services depends on the perception of quality of life after treatment. Late presentation of patients with PC is influenced by multiple factors not limited to sociocultural beliefs and taboos. We are recommending the use of a pragmatic approach to support the efforts of quality improvement in health service delivery for PC in Tanzania. More strategies on health education are required to reduce misconceptions about PC. Despite some effort on awareness campaigns through different platforms, more effort is needed in determining the causes of PC, the diagnostic/screening methods necessary for PC, timely health seeking, the available treatment options for PC, and the expected quality of life after treatment.

## Data Availability

The original contributions presented in the study are included in the article/supplementary material. Further inquiries can be directed to the corresponding author.
